# PI3K-AKT/mTOR Signaling in Psychiatric Disorders: A Valuable Target to Stimulate or Suppress?

**DOI:** 10.1093/ijnp/pyae010

**Published:** 2024-02-14

**Authors:** Yan Chen, Wei Guan, Mei-Lan Wang, Xiao-Yun Lin

**Affiliations:** Department of Neurology, Nantong Third People’s Hospital, Affiliated Nantong Hospital 3 of Nantong University, Nantong, Jiangsu, China; Department of Pharmacology, Pharmacy College, Nantong University, Nantong, Jiangsu, China; Department of Neurology, Nantong Third People’s Hospital, Affiliated Nantong Hospital 3 of Nantong University, Nantong, Jiangsu, China; Department of Neurology, Nantong Third People’s Hospital, Affiliated Nantong Hospital 3 of Nantong University, Nantong, Jiangsu, China

**Keywords:** Psychiatric disorders, PI3K-AKT, Depression, Targets, Signaling pathway, mTOR

## Abstract

Economic development and increased stress have considerably increased the prevalence of psychiatric disorders in recent years, which rank as some of the most prevalent diseases globally. Several factors, including chronic social stress, genetic inheritance, and autogenous diseases, lead to the development and progression of psychiatric disorders. Clinical treatments for psychiatric disorders include psychotherapy, chemotherapy, and electric shock therapy. Although various achievements have been made researching psychiatric disorders, the pathogenesis of these diseases has not been fully understood yet, and serious adverse effects and resistance to antipsychotics are major obstacles to treating patients with psychiatric disorders. Recent studies have shown that the mammalian target of rapamycin (mTOR) is a central signaling hub that functions in nerve growth, synapse formation, and plasticity. The PI3K-AKT/mTOR pathway is a critical target for mediating the rapid antidepressant effects of these pharmacological agents in clinical and preclinical research. Abnormal PI3K-AKT/mTOR signaling is closely associated with the pathogenesis of several neurodevelopmental disorders. In this review, we focused on the role of mTOR signaling and the related aberrant neurogenesis in psychiatric disorders. Elucidating the neurobiology of the PI3K-AKT/mTOR signaling pathway in psychiatric disorders and its actions in response to antidepressants will help us better understand brain development and quickly identify new therapeutic targets for the treatment of these mental illnesses.

## Graphical Abstract

**Graphical Abstract:**
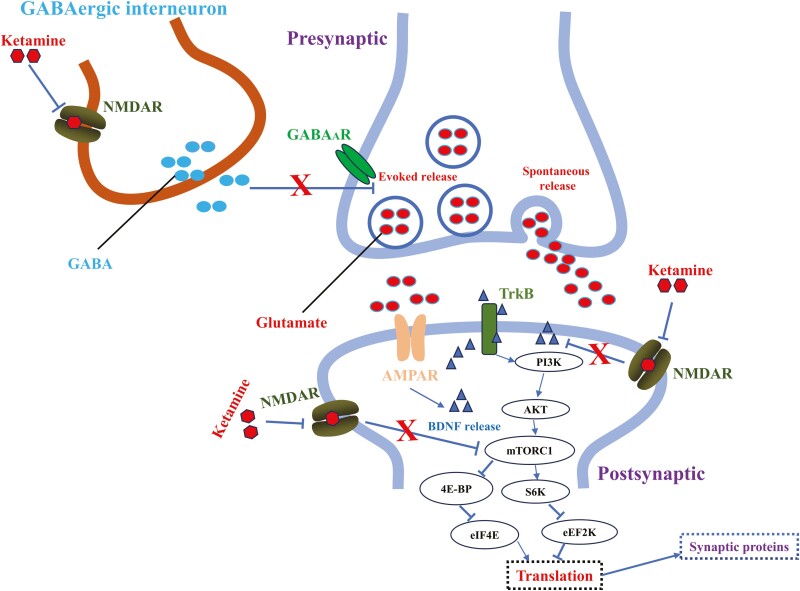

## INTRODUCTION

**Figure 1.**
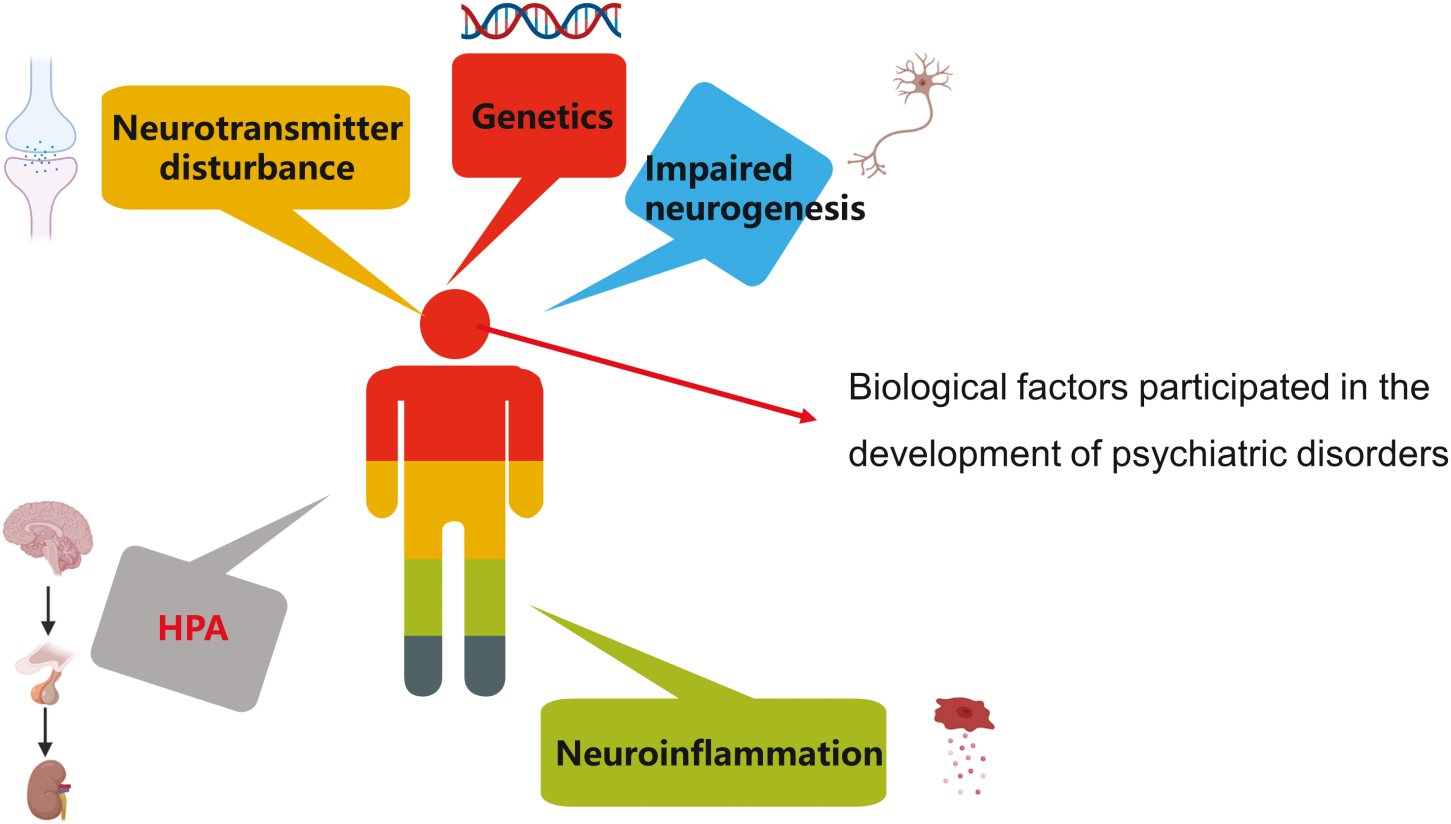
Biological factors participated in the development of psychiatric disorders.

Psychiatric disorders are a major global health problem, with extremely high rates of mortality and morbidity increasing annually. Globally, 970 million people suffered from psychiatric disorders in 2023, placing a heavy burden on society and respective families (Bernal-Vega et al., 2023). Clinical features of psychiatric disorders include emotional instability, cognitive disorders, and abnormal behavior (Table 1). Considering that public health nurses are a frequent first point of contact for those with depression, the nurses are key to depression detection and suicide prevention, especially in primary care settings (Hauenstein, 2003). Nurses can promote recovery from depression through psychoeducation, including encouraging a healthy lifestyle and enhancing social skills (Hauenstein, 2003). Previous research has implicated complex interactions between biological factors, psychologies, and social relationships in the pathogenesis of these disorders. However, the biological mechanisms and mechanistic pathways involved in psychiatric disorders have not been fully elucidated yet, leading to frequent misdiagnosis and incorrect treatment of patients in clinical practice. Recently, researchers have gained a deeper understanding of the biological factors, including genetic factors, neurotransmitter disturbances, hypothalamic-pituitary-adrenal axis overactivity, impaired neurogenesis, and neuroinflammation, involved in the development of psychiatric disorders (Figure 1) (Harrison et al., 2018; Villas Boas et al., 2019; Welcome, 2020). Numerous chemical drugs, such as fluoxetine, venlafaxine, levetiracetam, chlorpromazine, and olanzapine, have been developed based on these underlying biological mechanisms for the treatment of patients with psychiatric disorders (Scangos et al., 2023). However, drug toxicity and susceptibility, distressing adverse effects of antipsychotics, and cardiovascular and gastrointestinal adverse effects have been reported (Behlke et al., 2020; Oliva et al., 2021).

**Table 1.** Clinical Manifestations of Patients With Depression, Schizophrenia, and Epilepsy

Psychiatric and neurologic disorders	Clinical manifestation	References
Depression	Depressed mood most of the day	(Mohan et al., 2023)
	Markedly diminished interest or pleasure in activities most of the day	
	Somnipathy nearly every day and loss of weight	
	Diminished ability to think or concentrate	
	Suicidal thought	
Schizophrenia	Hallucinations	(Carpenter and Tandon, 2013)
	Delusions	
	Disorganized speech	
	Grossly disorganized or catatonic behavior	
	Diminished emotional expression or avolition	
Epilepsy	Epileptic seizures (tonic-clonic seizures)	(Peter and Camfield, 2013)
	Staring spells for 10–20 seconds	
	Febrile seizures	
	Nocturnal vomiting	
	Loss of speech and social interactions	

mTOR, a serine/threonine (Ser/Thr) kinase, is a common target in a broad range of pathological conditions. This kinase is an important intracellular signaling pathway component that regulates cell growth and metabolism (Karalis and Bateup, 2021). In addition, multiple signaling pathways and receptors, such as adenosine monophosphate-activated protein kinase (AMPK) and neurotrophic factors, converge to transmit information (Sun et al., 2018; Aleksandrova and Phillips, 2021), and mTOR activation promotes neural maturation, synapse formation, and synaptic plasticity (Banko et al., 2005; Xu et al., 2021). Abnormal activity of the mTOR pathway causes various neurological and psychotic disorders, including depression and schizophrenia (Chadha et al., 2021; Kim et al., 2022). Moreover, recent research has focused on the role of the mTOR pathway as a therapeutic target underlying the effects of newly developed antipsychiatric drugs, which are superior to classic antipsychotics in terms of rapid onset of the pharmacologic effect (Li, 2020). Therefore, mTOR is a promising target for the treatment of psychiatric and neurological disorders. In this review, we focus on describing the role of mTOR and the related pathways in depression, schizophrenia, and epilepsy and summarize specific antipsychotics that exert their pharmacologic effects via the activity of the mTOR signaling pathway.

## SEARCH STRATEGY AND SELECTION CRITERIA

**Figure 2.**
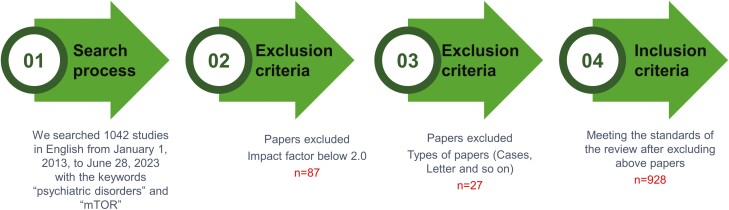
The screening process of articles about PI3K-AKT/mTOR signaling in psychiatric disorders.

We searched PubMed, Web of Science, Engineering Village 2, Wolters Kluwer, and search engines, such as Google and Safari, for publications in English from January 1, 2013, to June 28, 2023, using the keywords “psychiatric disorders,” “mTOR,” “depression,” “schizophrenia,” “epilepsy,” “signal pathway,” and “mechanisms.” Publications not written in English and articles in journals with impact factors below 2.0 points were excluded. Finally, certain clinical cases and conference reports pertinent to the theme of this review were incorporated into the discussion sections (Figure 2).

## STRUCTURE OF mTOR IN THE BRAIN

mTOR, a highly conserved Ser/Thr protein kinase located on chromosome 1 (1p36.22), is the catalytic component of 2 structurally distinct protein complexes, mTORC1 and mTORC2. mTOR is an atypical protein kinase that belongs to the phosphatidylinositol (PI) kinase-related kinase family (Keith and Schreiber, 1995; Hunter, 2014). mTOR was first discovered by Schreiber and Snyder in 1994 in yeast, and scientists have since discovered the mTOR protein in mammalian cells (Brown et al., 1994; Sabers et al., 1995).

**Figure 3.**
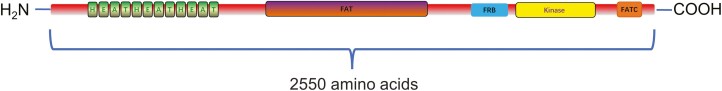
The structure of mTOR protein molecule.

The molecular weight of mTOR is 289 kDa, consisting of 2550 amino acids encoded by 7650 nucleotides (Murugan, 2019). The mTOR protein consists of multiple domains, HEAT repeats in its N terminus, a FRAP/ATM/TRRAP (FAT) domain in its middle position, and FKBP12-rapamycin binding and FATC domains in its C terminus (Figure 3) (Battaglioni et al., 2022).

**Figure 4.**
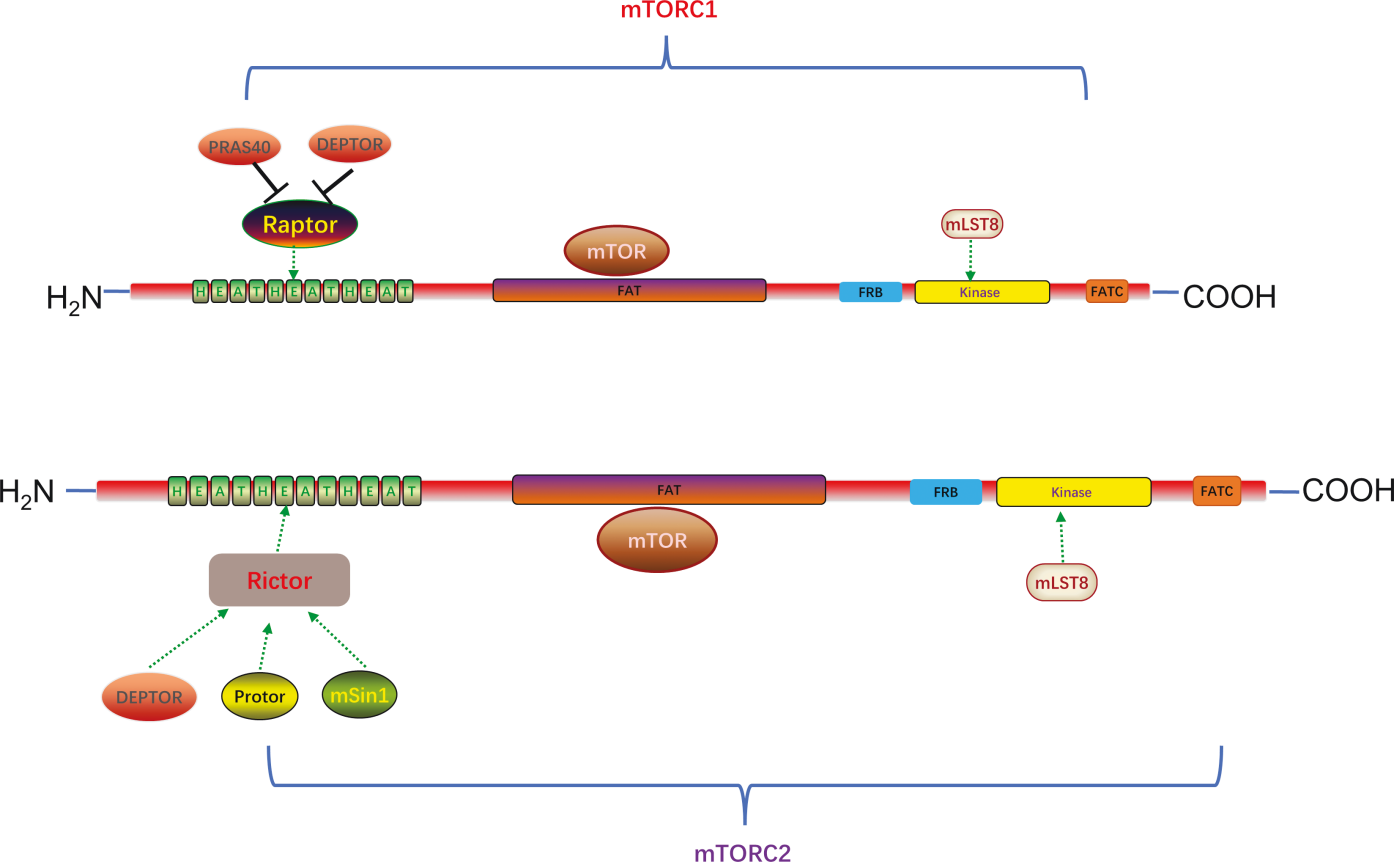
Domain structure of mTOR and components of mTORC1 and mTORC2.

In addition, mTORC1 and mTORC2 have 3 essential core subunits: the catalytic subunit mTOR, mLST8 (mammalian lethal with Sec13 protein 8), and Deptor (DEP domain-containing mTOR-interacting protein) (Yip et al., 2010; Chen et al., 2018). In addition to these shared proteins, mTORC1 and mTORC2 contain unique complex-specific proteins. PRAS40 (proline-rich Akt substrate 40 kDa) and Raptor (regulatory-associated protein of mTOR) are specific subunits of mTORC1, whereas mSin1 (mitogen-activated protein kinase-associated protein 1), Rictor (rapamycin-insensitive companion of mTOR), and Protor (protein observed with Rictor) are unique subunits of mTORC2 (Figure 4) (Popova and Jücker, 2021).

## mTOR SIGNALING IN PSYCHIATRIC DISORDERS

**Figure 5.**
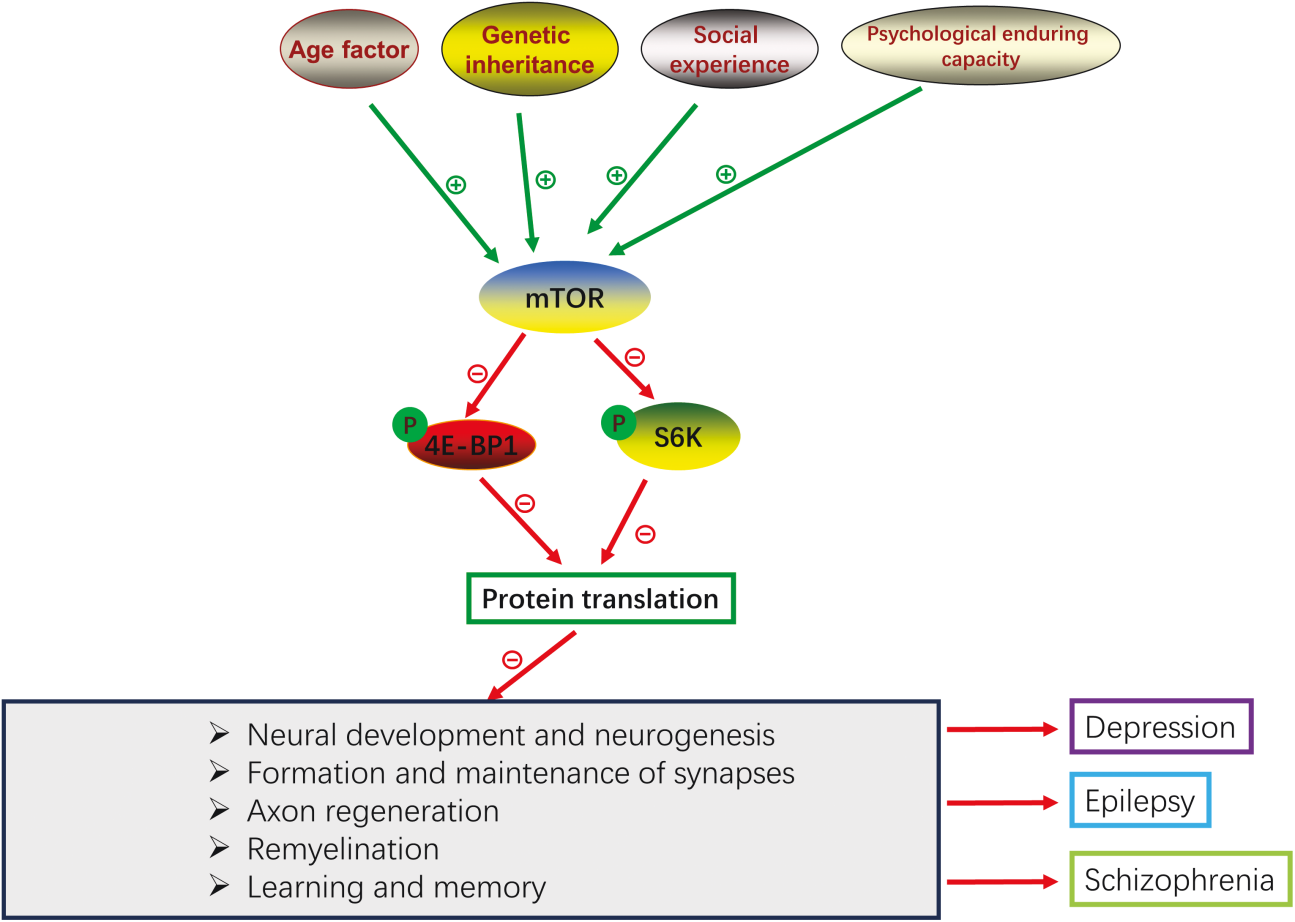
mTOR signaling pathway has a close relation with many physiological processes of the nervous system, including neural development and neurogenesis, formation and maintenance of synapses, axon regeneration, remyelination, learning and memory.

The activation of the mTOR signaling pathway is closely related to several physiological processes in the nervous system, including neural development and neurogenesis, formation and maintenance of synapses, axon regeneration, remyelination, learning, and memory (Figure 5) (Lipton and Sahin, 2014). mTOR dysfunction results in structural brain abnormalities associated with several psychiatric and neurological disorders, including depression, anxiety, schizophrenia, and epilepsy (Table 2) (Child and Benarroch, 2014). Therefore, pharmacological manipulation of mTOR signaling is a potentially important therapeutic target.

**Table 2.** Dysfunction of the mTOR Signalling Pathway in Psychiatric and Neurologic Disorders

Psychiatric and neurologic disorders	Samples	Pathway activity	Neurological manifestations	References
Anxiety	Hippocampus of high fat diet-induced obesity of mice	Upregulation	Mice showed depressive and anxiety-like behaviours	(Li et al., 2022)
Anxiety	Hippocampus of Dip2a KO mice	Downregulation	Anxiety-like behaviours in Dip2a KO mice	(Ma et al., 2023)
Anxiety	Hippocampus of maternal separation (MS) and chronic restraint stress (CRS)-induced mice	Downregulation	Anxiety-like behaviour and cognitive impairments	(Wang et al., 2020)
Anxiety	Hippocampus of S6K1^−/−^ mice	Downregulation	Anxiety-related behaviours	(Koehl et al., 2021)
Anxiety	The retinal tissue of CSDS-induced rats	Downregulation	Anxiety and depression-like behaviour	(Ma et al., 2022)
Depression	Hippocampus of LPS and CUMS-induced mice	Upregulation	Alleviated depression-like behaviours	(Shen et al., 2021)
Depression	Primary astrocytes from the cerebral tissues of the healthy rats induced by LPS	Upregulation	Depression-like behaviours	(Yang et al., 2023)
Depression	Blood samples of patients with depression	Upregulation	Alleviated depression-like behaviours	(Singh et al., 2023)
Depression	Dexamethasone (DEXA, 20 mg/kg)-induced depression in mice	Upregulation	Mice showed a substantial increase in immobility time	(Alhaddad et al., 2023)
Schizophrenia	Dorsolateral prefrontal cortex (DLPFC) from patients or frontal cortex of rats induced by haloperidol decanoate (28.5 mg/kg)	Downregulation	Schizophrenia	(Chadha and Meador-Woodruff, 2020)
Schizophrenia	Blood samples from patients with schizophrenia	Downregulation	Acute schizophrenia	(Cui et al., 2021)
Schizophrenia	DLPFC from patients	Downregulation	Schizophrenia accompanied by bipolar disorder	(Vanderplow et al., 2021)
Schizophrenia	PFC from patients or cortex from rats	Downregulation	Schizophrenia	(Ibarra-Lecue et al., 2020)
Epilepsy	Down-regulation	Alleviated epilepsy	mTOR and/or MAPK pathways	(Pernice et al., 2016)
Epilepsy	Down-regulation	Alleviated epilepsy	Signalling pathway AKT/mTOR	(Romero-Leguizamon et al., 2016)
Epilepsy	Up-regulation	Induced epilepsy	Dishevelled, Egl-10 and Pleckstrin (DEP) domain-containing protein 5 (DEPDC5)/ mTOR	(Jin et al., 2018)
Epilepsy	Up-regulation	Induced epilepsy	PI3K/Akt/mTOR signalling	(Duan et al., 2018)
Epilepsy	Down-regulation	Alleviated epilepsy	AKT/mTOR signalling pathway	(Gao et al., 2022)
Epilepsy	Down-regulation	Alleviated epilepsy	mTOR and 4E-BP-1	(Nguyen et al., 2022)
Epilepsy	Down-regulation	Alleviated epilepsy	Dynorphinm/TOR signalling pathway	(Liu et al., 2022)

### mTOR Signaling and Antidepressant Action

The mTOR signaling cascade plays important roles in depression and antidepressant drug activity, and activating or enhancing mTOR signaling has been demonstrated to have antidepressant effects in preclinical studies (Abdallah et al., 2020).

Depression is a common mental health disorder with high pathogenicity and fatality rates and has a strong influence on health problems, activities of daily life, and the work of an individual. The pathophysiology of depression is attributed to impaired neurogenesis and disruptions in neuroplasticity (Kandola et al., 2019).

mTOR, a Ser/Thr kinase, is a critical integrator in the development of the central nervous system and is involved in various processes, such as neuronal activity and axonal growth (Ma et al., 2021). Therefore, in this review, we have highlighted research outlining the underlying mechanisms of mTOR signaling in depression and the relationship between mTOR signaling and antidepressant drugs (Table 3).

**Table 3.** Role of the mTOR Signalling Pathway in Depression

Psychiatric disorders	Samples	Pathway activity	Effects on depression	Effect targets	References
Depression	Hippocampus and PFC in LPS or CUMS-induced mice	Upregulation	Alleviated depression	The Akt/mTOR pathway	(Shen et al., 2021)
Depression	Hippocampus of high-fat diet-induced mice	Upregulation	Induced depression	The AMPK/mTOR pathway	(Li et al., 2022)
Depression	Hippocampus and mPFC of CUMS and CSDS-induced mice	Downregulation	Induced depression	p-mTORC1, p-4E-BP-1 and p-p70S6K	(Wang et al., 2020)
Depression	Brain tissues of high-fat diet and CUMS-induced mice or LPS-induced astrocytes	Upregulation	Induced depression	PI3K/Akt/mTOR pathway	(Yang et al., 2023)
Depression	Hippocampus of CRS-induced mice or LPS-induced astrocytes	Upregulation	Alleviated depression	PI3K/Akt/mTOR pathway	(Li et al., 2022)
Depression	Prefrontal cortex in chronic kidney disease (CKD) mice	Downregulation	Induced depression	mTORC1-S6K pathway	(Wang et al., 2019)
Depression	Hippocampus of postpartum depression (PPD) rats	Upregulation	Alleviated depression	The TSPO and BDNF‑mTOR pathways	(Chen et al., 2022)
Depression	Hippocampus of clomipramine (CL)-treated rats	Downregulation	Induced depression	Phospholipase D (PLD)-mTOR signalling	(Feng and Huang, 2013)
Depression	Hippocampus of corticosterone-induced mice	Upregulation	Alleviated depression	mTOR/GSK3β pathway	(Wang et al., 2023)

Wang et al. (2022) reported that mTOR phosphorylation was markedly decreased in the prefrontal cortex of chronic unpredictable mild stress (CUMS)-induced mice compared with that in the control group. The CUMS depression mouse model was successfully established using behavioral tests, including the tail suspension test (TST), forced swimming test (FST), and open field test. Additionally, the accumulated immobility time in the FST and TST was significantly reduced in CUMS-induced mice after crocin treatment, which significantly increased mTOR phosphorylation. Furthermore, rapamycin administration via lateral ventricle injection into the prefrontal cortex of these mice reversed the protective effect of crocin on depressive behavior in mice subjected to CUMS, which was associated with decreased mTOR phosphorylation levels (Wang et al., 2022). Similarly, decreased mTOR phosphorylation was observed in rat microglia treated with lipopolysaccharide (LPS). Interestingly, daily Shugan granule (SGKL) treatment at a dose of 0.63 g/kg significantly abrogated depression-like behaviors in chronic restraint stress (CRS)‐induced rats by reducing inflammatory cytokine levels in the hippocampus and colon. Furthermore, SGKL treatment altered gut microbiota, which was considered to have a direct link on behavior in the hippocampus of CRS‐evoked rats, and the antidepressant effect of SGKL was mediated by the activation of the mTOR pathway in LPS‐stimulated rat microglia. However, LY294002, a PI3K inhibitor, significantly blocked the protective effects of SGKL on LPS-stimulated microglia, which was accompanied by decreased mTOR activation (Li et al., 2022). In conclusion, the mTOR signaling pathway is a crucial target for developing new antidepressant drugs.

mTOR phosphorylation level in animal models of simulated depression is equivocal because some studies have reported that chronic stress promotes mTOR phosphorylation, thereby increasing the expression level of phosphorylated mTOR in the hippocampus and mPFC of rodents, eventually leading to depressive behaviors (Li et al., 2022; Yang et al., 2023). As observed by Alhaddad et al. (2023), mice that received dexamethasone (DEXA; 20 mg/kg) for 21 days exhibited depressive behaviors along with increased levels of glucose transporter 1 and 3 (GLUT1 and GLUT3), glycolytic enzymes (hexokinase and pyruvate kinase), and mTOR activity in the hippocampus and frontal cortex compared with control mice. However, these effects were significantly reversed in mice after treatment with 10 or 30 mg/kg DEXA for 21 days (Alhaddad et al., 2023). Consistent with these previous studies, Yang et al. (Yang et al., 2023) also indicated that *Morinda officinalis* oligosaccharides relieved depression-like behaviors and displayed a protective effect against brain injury in rats that received high salt and CUMS. Through in vitro experiments, they determined that the activation of the mTOR pathway was inhibited, and the formation of autophagosomes and autolysosomes was increased in LPS-stimulated astrocytes after *Morinda officinalis* oligosaccharide administration via targeting and elevating mitofusion 2 expression. These studies provide convincing evidence that reducing mTOR activity can enhance protective autophagy and may ameliorate depression in mice.

**Figure 6.**
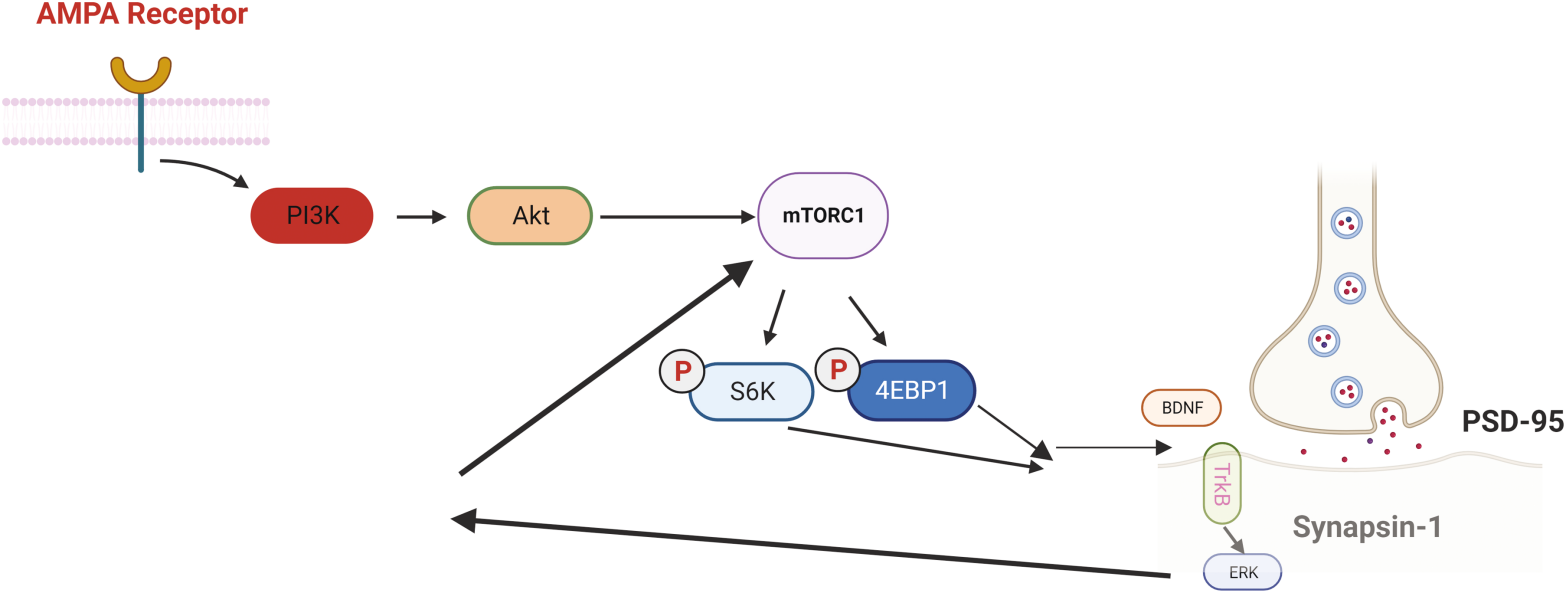
Neuroplasticity as a convergent mechanism for antidepressants. Specifically, antidepressants induced AMPAR activation that in turn potentiated BDNF-TrkB and mTOR signaling, thus upregulating the expression of neuroplasticity-related genes and protein synthesis of synaptic components.

In addition, mTOR signaling is involved in the antidepressant effects of some clinical antidepressants used as the first choice for treating patients with depression, including ketamine, escitalopram, paroxetine, and tranylcypromine (Table 4) (Zanos and Gould, 2018; Pham and Gardier, 2019). Therefore, the antidepressant effects of these drugs seem to be increasingly associated with the blockade of N-methyl-D-aspartate (NMDA) receptors located on inhibitory gamma-aminobutyric acid (GABA)-ergic neurons. The subsequent increase in glutamate promotes the release of BDNF, thereby increasing protein synthesis in neuronal dendrites (Deutschenbaur et al., 2016). The activation of these receptors by α‐amino‐3‐hydroxyl‐5‐methyl‐4‐isoxazole‐propionate (AMPA) and BDNF further activates downstream intracellular mTOR signaling, triggering the translational machinery and leading to neuroprotection and neurogenesis (Figure 6) (Li et al., 2010; Duman et al., 2012).

**Table 4.** mTOR signalling pathway involvement in antidepressant effects

Antidepressant or other drugs	Pathway activity	Effects on depression	Effect targets	References
Paroxetine	Up-regulation	Alleviated depression	Hippocampal mTOR signalling	(Xu et al., 2018)
Ketamine	Up-regulation	Alleviated depression	The AMPK/mTOR/BDNF pathway	(Li et al., 2010; Zhou et al., 2014)
Rosiglitazone	Down-regulation	Alleviated major depressive disorder (MDD)	pAKT/p38MAPK/mTOR/4EBP1 pathway	(Alhaddad et al., 2023)
Fluoxetine	Up-regulation	Reduced depression	mTOR, p70S6K and 4E-BP-1, PSD-95 and synapsin I	(Liu et al., 2015)
Sertraline	Up-regulation	Induced depression	AMPK-MTOR signalling	(Hwang et al., 2021)
Rapastinel	Down-regulation	Induced depression	ERK/mTOR/VGF/BDNF/TrkB signalling	(Shen et al., 2022)
Fluvoxamine	Up-regulation	Alleviated depression	mTOR signalling	(Xu et al., 2020)
CP-101, 606	Down-regulation	Induced depression	mTOR signalling	(Qin et al., 2023)
Atorvastatin	Up-regulation	Alleviated depression	PI3K/Akt/GSK-3β and mTOR	(Ludka et al., 2016)
Escitalopram, paroxetine, and tranylcypromine	Up-regulation	Alleviated depression	Phospho-mTOR and its down-stream regulators phospho-4E-BP-1 and phospho-p70S6K	(Park et al., 2014)
AZD6765	Up-regulation	Alleviated depression	PI3K/Akt/mTOR/GSK3β	(Neis et al., 2020)
Agmatine	Up-regulation	Alleviated depression	AMPA receptors and mTOR signalling	(Neis et al., 2016)
Scopolamine	Up-regulation	Scopolamine -induced antidepressant activity	Scopolamine-induced synaptic plasticity and mTOR pathway activation via protein kinase A (PKA) pathway	(Dong et al., 2018)
Scopolamine	Up-regulation	Antidepressant-like effects of scopolamine	AMPA and mTOR signalling pathways	(Martin et al., 2017)
(2R,6R)-HNK	Activation	Antidepressant properties	AMPAR, mTOR and BDNF signalling	(Cavalleri et al., 2018)
(2R,6R)-HNK	Activation	Antidepressant action	Activation of the mTOR pathway and BDNF	(Herzog et al., 2021)

**Figure 7.**
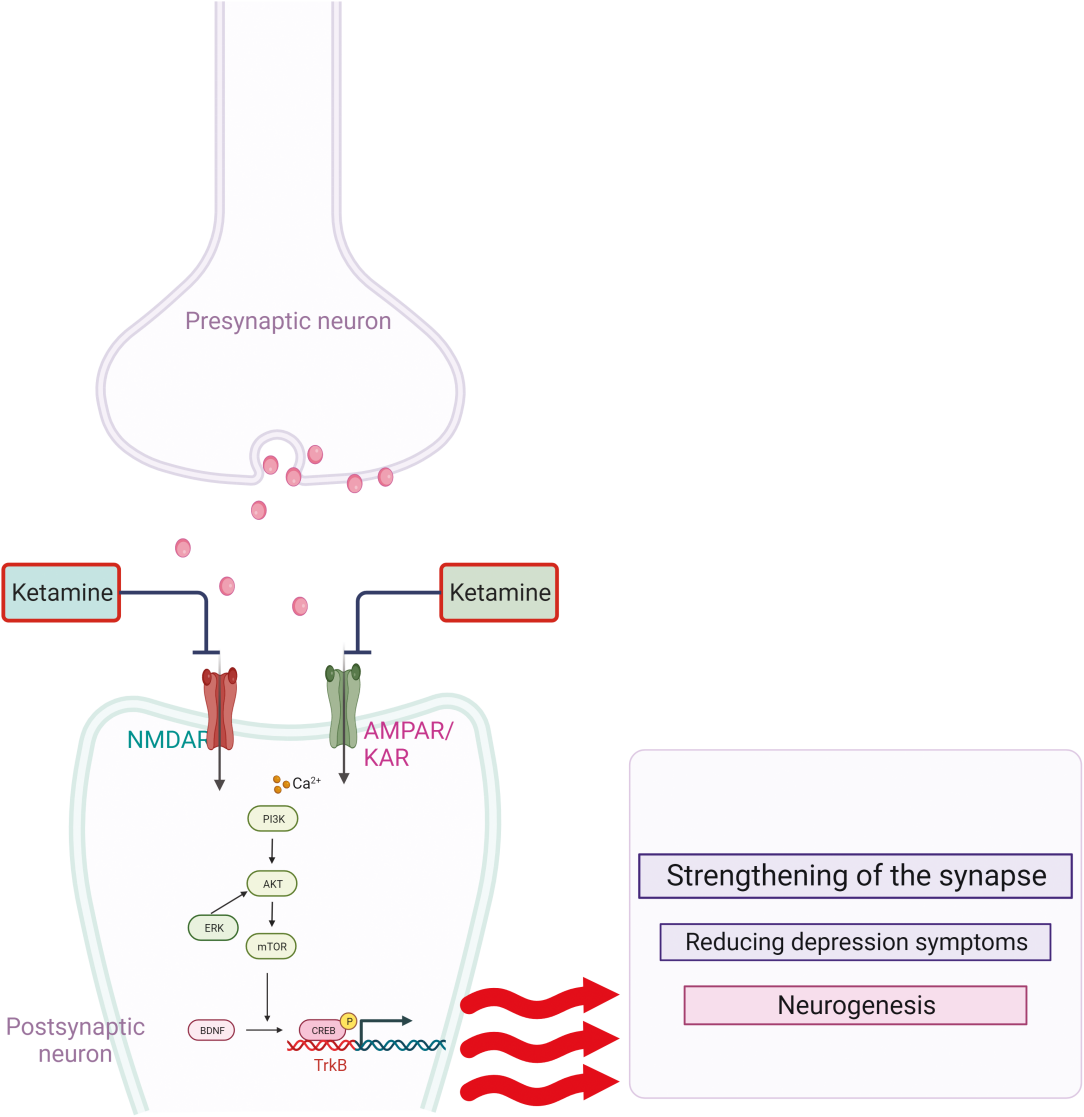
The effects of AMPA receptor modulators on mTOR and BDNF expression during the procedure of ketamine exerting antidepressant effects.

Clinical observations have shown that ketamine, at certain doses, can relieve depressive symptoms in patients with robust deficits in mTOR signaling in the prefrontal cortex (Jernigan et al., 2011). Preclinical studies have reported that the rapid antidepressant response to ketamine and paroxetine is mediated via the activation of the mTOR pathway in animal models of depression (Li et al., 2010; Xu et al., 2018). Furthermore, Rafało-Ulińska et al. (2022) demonstrated that mTOR expression was decreased in the prefrontal cortex of mice exposed to chronic unpredictable stress (Rafało-Ulińska and Pałucha-Poniewiera, 2022). Moreover, the immobility time of rats during the FST decreased rapidly after ketamine treatment, and researchers also observed an increase in the phosphorylation levels of mTOR and BDNF in the hippocampus and prefrontal cortex of rats (Figure 7) (Zhou et al., 2014). Tang et al. (2015) showed that ketamine produced rapid antidepressant effects by increasing the phosphorylation of the mTOR effectors 4E-BP1 and p70S6K, downstream targets, such as the synaptic protein GluR1, and upstream regulators, including ERK and Akt (Tang et al., 2015).

Similarly, other antidepressant treatments promote antidepressant behavioral responses via the activation of the PI3K/Akt/mTOR signaling pathway (Figure 7) (Li et al., 2010). For example, Ludka et al. (2016) demonstrated that atorvastatin exerted an antidepressant-like effect in mice via the activation of Akt and mTOR and inhibition of GSK-3β (Ludka et al., 2016). Liu et al. (2015) showed that chronic fluoxetine treatment reversed the decreased expression of mTOR, p70S6K, 4E-BP-1, PSD-95, and synapsin I in the hippocampus and amygdala of CUMS-induced mice (Liu et al., 2015). Xu et al. (2018, 2020) demonstrated that hippocampal mTOR signaling is required for the antidepressant effects of fluvoxamine and paroxetine (Xu et al., 2018, 2020). In contrast, several studies have suggested that imipramine exerts its effects by inhibiting PI3K/Akt/mTOR signaling in the human glioma cell line U-87MG or hippocampus of rats (Jeon et al., 2011; Pottoo et al., 2021).

The metabolism of ketamine to (2S,6S;2R,6R)-hydroxynorketamine (HNK) is essential for its antidepressant effects (Hess et al., 2022). In addition, (2R,6R)-(HNK) exerted antidepressant actions in vivo via its neuroactive effects and sustained AMPA receptor activation, rather than NMDAR inhibition (Zanos et al., 2016), whereas it had fewer adverse effects than ketamine. These studies suggest a novel mechanism underlying the antidepressant properties of ketamine, which are principally associated with the pharmacological properties of its metabolites.

Therefore, understanding the molecular mechanisms underlying mTOR signaling is necessary to identify novel therapeutic targets for antidepressant drug development.

### mTOR Signaling in Epilepsy

Several preclinical and clinical studies have revealed that mTOR signaling may be a crucial target in epileptogenesis. mTOR levels are upregulated in human tissue studies of acquired epilepsy or seizures (Ostendorf and Wong, 2015). An overstimulation of mTOR signaling leads to abnormalities in brain development, thereby contributing to epileptogenesis (Iori et al., 2016). In this review, we summarize the relationship between the mTOR signaling pathway and epileptogenesis (Table 2).

Epilepsy is a heterogeneous disorder characterized by persistent (spontaneous) or intermittent epileptic seizures caused by genetic and acquired etiologies (Löscher et al., 2020). Abnormal activity in cortical neurons is the main pathophysiological mechanism underlying epileptogenesis (Neri et al., 2022). Moreover, epilepsy is a comorbidity of a large variety of neurological disorders, including depression and neurodegenerative diseases (Kanner and Bicchi, 2022).

The activation of the mTOR pathway regulates synaptic plasticity and neurogenesis, which indirectly influences neuronal excitability (Wong, 2013). Based on these studies, the mTOR pathway may be involved in the development of epilepsy under pathological conditions. Furthermore, hyperactive mTOR signaling is found in animal models of epilepsy and the postmortem cerebral cortex of patients with epilepsy (Ostendorf and Wong, 2015). Specifically, the activity of the PI3K/Akt/mTOR signaling pathway is upregulated in the temporal and frontal cortices of patients with epilepsy or recurrent seizures (Liu et al., 2014; Lin et al., 2015). Moreover, preliminary clinical studies have shown that the administration of mTOR inhibitors, such as rapamycin and everolimus, reduces the intensity and frequency of seizures (Table 5) (Marsan and Baulac, 2018).

**Table 5.** mTOR Signalling Pathway Involvement in Antiepileptic Drug Effects

Antiepileptic drugs	Pathway activity	Effects on depression	Effect targets	References
Everolimus	Down-regulation	Alleviated epilepsy	mTOR	(Krueger et al., 2013)
Everolimus	Down-regulation	Alleviated epilepsy	mTOR	(Franz et al., 2018)
Everolimus	Down-regulation	Alleviated epilepsy	PI3K/Akt/mTOR and NF-kB/IL-6 signalling	(Huang et al., 2021)
Rapamycin	Down-regulation	Alleviated epilepsy	mTOR signalling	(Zhao et al., 2020)
Rapamycin	Down-regulation	Alleviated epilepsy	mTOR, p70S6K and 4E-BP-1	(Iffland et al., 2022)
Rapamycin	Down-regulation	Alleviated epilepsy	mTOR and P-glycoprotein (P-gp)	(Chi et al., 2017)
Rapamycin	Down-regulation	Alleviated epilepsy	mTOR, p70S6K and 4E-BP-1	(Wang et al., 2018)

In several animal models, researchers have found pathological mTOR hyperactivation, whereas mTOR inhibitors abrogate the progress of epileptic seizures. For example, mTOR activity significantly increased in the hippocampus of pilocarpine-treated rats, whereas rapamycin and SB-399885 suppressed epileptic seizures in rats by decreasing mTOR activity and inhibiting 5-HT6 receptor expression (Wang et al., 2015). Additionally, PI3K-Akt-mTOR signaling was hyperactivated in the hippocampus of neuronal subset-specific phosphatase and tensin homologue knockout mice, whereas rapamycin treatment normalized Kv1.1 protein levels in neuronal subset-specific phosphatase and tensin homologue knockout mice and dysfunction in Kv1 channels related to epilepsy, suggesting that mTOR signaling regulates voltage-gated ion channel expression in a mouse model of epilepsy (Nguyen and Anderson, 2018).

Emerging evidence suggests that the dysregulation of the mTOR signaling pathway can lead to the upregulation and secretion of various cytokines (TNFα, IL-1, IFNγ, IL-6) and chemokines (MCP-1/CCL2, CCL3, CCL5) in the brain tissue samples from patients with intractable epilepsy (Arisi et al., 2015; Uludag et al., 2015). This indicates that mTOR signaling and neuroinflammation interact in epilepsy. For instance, the inflammatory response induced by IL-1β increased seizure susceptibility and was involved in the pathogenesis of mesial temporal lobe epilepsy via the PI3K/Akt/mTOR signaling pathway (Xiao et al., 2015). Moreover, animal experiments have demonstrated that drugs inhibiting inflammatory responses and mTOR activation prevented apoptotic neuronal death in the hippocampus of mice against status epilepticus (supplementary Figure 1) (Park et al., 2022).

Therefore, exploring the key regulatory signaling pathways involved in epilepsy is of great importance, and inhibition of the mTOR signaling pathway could be a promising therapeutic avenue against the development of epilepsy.

### mTOR Signaling in Schizophrenia

Schizophrenia is a serious mental disorder characterized by synaptic disruption during neurodevelopment and detrimentally affects a significant portion of the global population. Various extrinsic risk factors, such as stress, dietary habits, and drug abuse, may contribute to the occurrence of schizophrenia.

A dysfunction in the activity of the mTOR signaling pathway during neurodevelopment caused by social and environmental factors may alter dendritic spine morphology, leading to the impairment of synaptic plasticity and neurogenesis and thereby increasing the risk of developing schizophrenia. Therefore, in this review, we discuss the functional roles of the mTOR signaling cascade in the pathogenesis of schizophrenia and provide a theoretical foundation for the development of novel therapeutic targets based on mTOR signaling (Table 6).

**Table 6.** Abnormal mTOR Expression in Schizophrenia

Psychiatric disorders	Pathway activity	Effects on depression	Effect targets	References
Schizophrenia	Upregulation	Alleviated schizophrenia	miR-144-3p/ATP1B2/mTOR signalling pathway	(Pan et al., 2022)
Schizophrenia	Upregulation	Alleviated schizophrenia	NRG1/ErbB4 and PI3K/AKT/mTOR signalling pathways	(Nawwar et al., 2022)
Schizophrenia	Upregulation	Induced schizophrenia	Akt/mTOR pathway	(Ibarra-Lecue et al., 2018)
Schizophrenia	Upregulation	Induced schizophrenia	Akt/mTOR pathway	(Kim et al., 2009)
Schizophrenia	Downregulation	Induced schizophrenia	miR-137/ neuregulin (Nrg)/ErbB and BDNF/PI3K-Akt-mTOR	(Thomas et al., 2017)
Schizophrenia	Downregulation	Induced schizophrenia	Protein kinase B gamma (PKBγ)/AKT3 and AKT/mTORC2 signalling	(Howell et al., 2017)
Schizophrenia	Upregulation	Alleviated schizophrenia	GluN2B-NMDARs and the mTOR pathway	(Iafrati et al., 2014)

Under normal physiological conditions, mTOR signaling plays a pivotal role in synaptic plasticity and facilitates long-term memory formation (Martin et al., 2000). However, the disruption of mTOR activity may result in the development of schizophrenia. Recently, Chadha et al., (2021) revealed that the expression and phosphorylation levels of mTOR were decreased in the posthumous brain specimens of patients with schizophrenia. Furthermore, downstream regulators of mTOR complex signaling, such as ribosomal protein S6, are reduced in postmortem schizophrenic brains. Additionally, they found that the administration of rapamycin in postmortem brain tissue significantly suppressed mTOR activity via an increase in AMPK signaling compared with that in normal samples (Chadha et al., 2021). Overall, this indicates that disrupted mTOR signaling impairs neurodevelopment and ultimately leads to an increased risk of schizophrenia.

Chadha and Meador-Woodruff further showed a reduction in AKT-mTOR expression or mTOR phosphorylation in dorsolateral prefrontal cortex tissues in postmortem brain samples of people with schizophrenia (Chadha and Meador-Woodruff, 2020). The AKT-mTOR signaling pathway is closely associated with neural plasticity and neurotransmission, which have been implicated in the pathophysiology of schizophrenia (Fortin et al., 2012).

Although most studies have shown that mTOR signaling is downregulated in patients with schizophrenia, conflicting results from Izumi et al. (2022) indicated that mTOR expression was elevated in the prefrontal cortex, whereas the expression level of the effector of mTOR translational regulation (phospho-S6) was decreased in the superior temporal gyrus and prefrontal cortex of postmortem patients with schizophrenia (Izumi et al., 2022). Because this result contradicts previous studies, we speculate that the main cause of this phenomenon was mTOR signaling variation in distinct brain regions, which was confirmed by another study (Gururajan and van den Buuse, 2014).

In animal models of simulated schizophrenia, enhanced phosphorylation levels of PI3K, Akt, and mTOR relieved abnormalities in schizophrenia-like behaviors in rodents (Nawwar et al., 2022). Pan et al. (2022) observed that the downregulated miR-144-3p and upregulated expression of the beta2-subunit of Na(+)/K(+)-ATPase (ATP1B2)/ PI3K/Akt/mTOR signaling pathway abrogated neuronal cell damage and schizophrenia-like behavioral abnormalities (anxiety and recognition memory deficits, weakened motor coordination, impaired spatial memory, and swimming ability) in MK-801–induced rats in a model of schizophrenia (Pan et al., 2022). Recently, Nawwar et al. (2022) showed that antipsychotic drugs, such as sertindole (2.5 mg/kg, orally) or aripiprazole (3 mg/kg, orally), significantly ameliorated schizophrenic-like behavior (locomotor activity impairments, cognitive deficits, shorter total interaction duration, and reduced sucrose preference) in ketamine-induced rats via the activation of the PI3K/AKT/mTOR signaling pathway and increase in the mRNA expression of neuregulin1 (NRG1) and epidermal growth factor receptor-4 in the hippocampus of rats with schizophrenia (supplementary Figure 2)(Nawwar et al., 2022). Further, aripiprazole and sertindole alleviated the positive and negative symptoms of schizophrenia and partially improved cognitive dysfunction without producing extrapyramidal side effects (Gupta and Masand, 2004; Hereta et al., 2020). These studies highlighted that the neuroprotective effects of aripiprazole and sertindole may be mediated through the PI3K/AKT/mTOR signaling pathway in rats with ketamine-induced schizophrenia. Furthermore, Ju et al. (2020) showed that mogroside V treatment reduced MK-801–induced prepulse inhibition and social withdrawal in mice with schizophrenia, and its metabolite 11-oxo-mogrol promoted neurite outgrowth and inhibited cellular apoptosis by reversing the inactivation of the AKT and mTOR phosphorylation induced by MK801 in primary neuronal cell cultures obtained from the prefrontal cortex of pregnant mice (Ju et al., 2020).

However, the role of the PI3K/AKT/mTOR signaling pathway in schizophrenia remains controversial. An overexpression or increased functionality of the Akt/mTOR signaling pathway predisposes individuals to schizophrenia. In a study by Ibarra-Lecue et al. (2018), activation of the Akt/mTOR pathway exacerbated schizophrenia-like responses in a tetrahydrocannabinol-induced mouse model of schizophrenia (10 mg/kg i.p.). The sensitivity of mice to tetrahydrocannabinol-induced schizophrenia-like effects was demonstrated to be inhibited by rapamycin treatment (Ibarra-Lecue et al., 2018). In addition, the pharmacological inhibition of the AKT-mTOR signaling pathway rescues developmental defects in new neurons in the hippocampus of adult mice.

In summary, schizophrenia is a chronic psychotic disorder characterized by positive and negative symptoms. However, its neural basis remains poorly understood. To date, relatively few studies have examined the effects of mTOR signaling on the positive and negative symptoms of schizophrenia. We have speculated that the AKT-mTOR signaling pathway is either a suppressor or promoter involved in schizophrenia. Future studies should be performed to investigate the contribution of this pathway more comprehensively.

## CONCLUSIONS AND FUTURE PROSPECTS

mTOR, a Ser/Thr kinase, is ubiquitously expressed in the human brain and orchestrates a series of processes, such as cell growth and metabolism, by sensing and integrating several intracellular and environmental cues. Accumulating evidence suggests that the mTOR signaling cascade regulates numerous neuronal processes, from neurodevelopment and synaptic plasticity to neuronal apoptosis (Lipton and Sahin, 2014). Under normal conditions, mTOR is involved in regulating numerous physiological functions, and dysfunction of these physiological processes may contribute to the pathogenesis of several psychiatric and neurological disorders, including depression, epilepsy, and schizophrenia. In this review, we have discussed the mechanisms and causal roles of mTOR hyperactivation in several mental disorders. In addition, our review highlights recent advances about the role of the mTOR signaling pathway as an enhancer or inhibitor involved in the mechanisms of action of antipsychotics, such as ketamine, everolimus, and olanzapine.

Moreover, we have also discussed research indicating that the dysregulation of the mTOR signaling cascade is associated with neurodevelopmental disorders, which would, in turn, lead to depression. The activation of the PI3K-Akt-mTOR signaling pathway contributes to the neurodevelopment of the cerebral neocortex and alleviates depression-like phenotypes. These theoretical foundations have great potential for identifying new therapeutic targets for depression.

Recently, clinical observations have shown that the antidepressant effects of some antidepressants, such as ketamine, require the activation of the mTOR pathway (Nowak et al., 2019). The antidepressant effects of ketamine are dependent on the inhibition of NMDA receptors and activation of glutamate-AMPA receptors via enhanced BDNF release, activation of the TrkB receptor and PI3K/Akt, and subsequent activation of mTORC1. These research findings indicate that mTOR signaling may be involved in the antidepressant effects of ketamine; however, this is controversial. Abdallah et al. (Abdallah et al., 2020) highlighted data that pretreatment with oral rapamycin (6 mg) in patients with major depressive disorder failed to block and prolonged the antidepressant effects of ketamine, which further confirmed that the inhibition of ketamine, and not the peripheral administration of rapamycin, followed the intracortical effects in animal models (Autry et al., 2011). The antidepressant effects of ketamine are prolonged by rapamycin via enhancing autophagy, and the anti-inflammatory effects of rapamycin protect synapses (Abdallah et al., 2020). Additionally, Yang et al. (2018) showed that mTOR played a role in the antidepressant effects of (*S*)-ketamine, but not of (*R*)-ketamine, suggesting that the activation of mTOR signaling is necessary for the antidepressant actions of only (*S*)-ketamine (Yang et al., 2018). In contrast, several nonclinical studies indicated that ketamine had no strong effects on mTORC1 signaling (Popp et al., 2016). A meta-analysis conducted by Averill et al. (Averill et al., 2022) revealed that no significant effects exist for rapamycin pretreatment on suicidal ideation, indicating that the anti-suicidal effects of ketamine are independent from its antidepressant effects. In addition, the role of mTOR in the activity of other antidepressants is controversial. Acute vortioxetine, but not fluoxetine or ketamine, transiently increased mTOR expression in the frontal cortex of rats (du Jardin et al., 2016). Liu et al. (2015) reported that chronic fluoxetine treatment attenuated the CUMS-induced mTOR phosphorylation reduction in the hippocampus and amygdala of mice, but not in the frontal cortex or the hypothalamus, indicating that fluoxetine regulated mTOR signaling in a region-dependent manner in depression-like mice (Liu et al., 2015). However, further in vivo and in vitro studies are required to elucidate the mechanism of action of ketamine as an antidepressant.

Additionally, prior research has indicated that the mTOR pathway is hyperactive during epileptogenesis in the cerebral cortex of humans with epilepsy or in animal models. Furthermore, several preclinical studies have shown that rapamycin administration decreases chronic seizures by decreasing neuronal excitability. Overall, mTOR inhibitors have antiepileptic effects in patients with epilepsy. However, controlled trials are needed to determine the optimal conditions, timing, and dosage of therapy. Although numerous PI3K/AKT/mTOR pathway inhibitors have been extensively studied, their potential adverse effects, such as severe hepatotoxicity and pneumonitis, have largely restricted the clinical application of these inhibitors (Zhang et al., 2019). However, the mechanisms underlying these toxicities remain unclear, and future research is needed to better understand PI3K/AKT/mTOR inhibitor-induced toxicities.

Finally, the mTOR signaling pathway was downregulated in the schizophrenic brain. We further discussed whether disrupted AKT-mTOR signaling was associated with schizophrenia pathophysiology and abnormal neuronal morphology. However, overactivation of mTOR signaling might increase weight gain, diabetes, and other metabolic complications (Zhuo et al., 2022). In addition, excessive nutrient intake promotes mTOR signaling, which in turn leads to kidney disease and cancer (Jia et al., 2014). Future studies are needed to address the challenges regarding the side effects of antipsychotic responses.

In conclusion, this systematic and comprehensive review has focused on the critical role of mTOR signaling in psychiatric disorders and the effects of antipsychotic medications. These findings may be used to develop novel drugs that serve as agonists or inhibitors of the mTOR signaling pathway, potentially paving the way for innovative treatments of psychiatric disorders and raising awareness among doctors and nurses of this debilitating condition and its serious social hazardous and suggesting interventions that can bring about improvement.

## Supplementary Material

pyae010_suppl_Supplementary_Material

pyae010_suppl_Supplementary_Figure_S1

pyae010_suppl_Supplementary_Figure_S2

## Data Availability

Not applicable.
