# Supplementary material for: PI3K-AKT/mTOR Signaling in Psychiatric Disorders: A Valuable Target to Stimulate or Suppress?
Source: Int J Neuropsychopharmacol. 2024 Feb 14;27(2):pyae010. doi: 10.1093/ijnp/pyae010 (PMC10888523; doi:10.1093/ijnp/pyae010)

**Fig. S1** Antiepileptic drugs inhibiting inflammatory responses and mTOR activation prevented apoptotic neuronal death in the hippocampus of mice against status epilepticus.


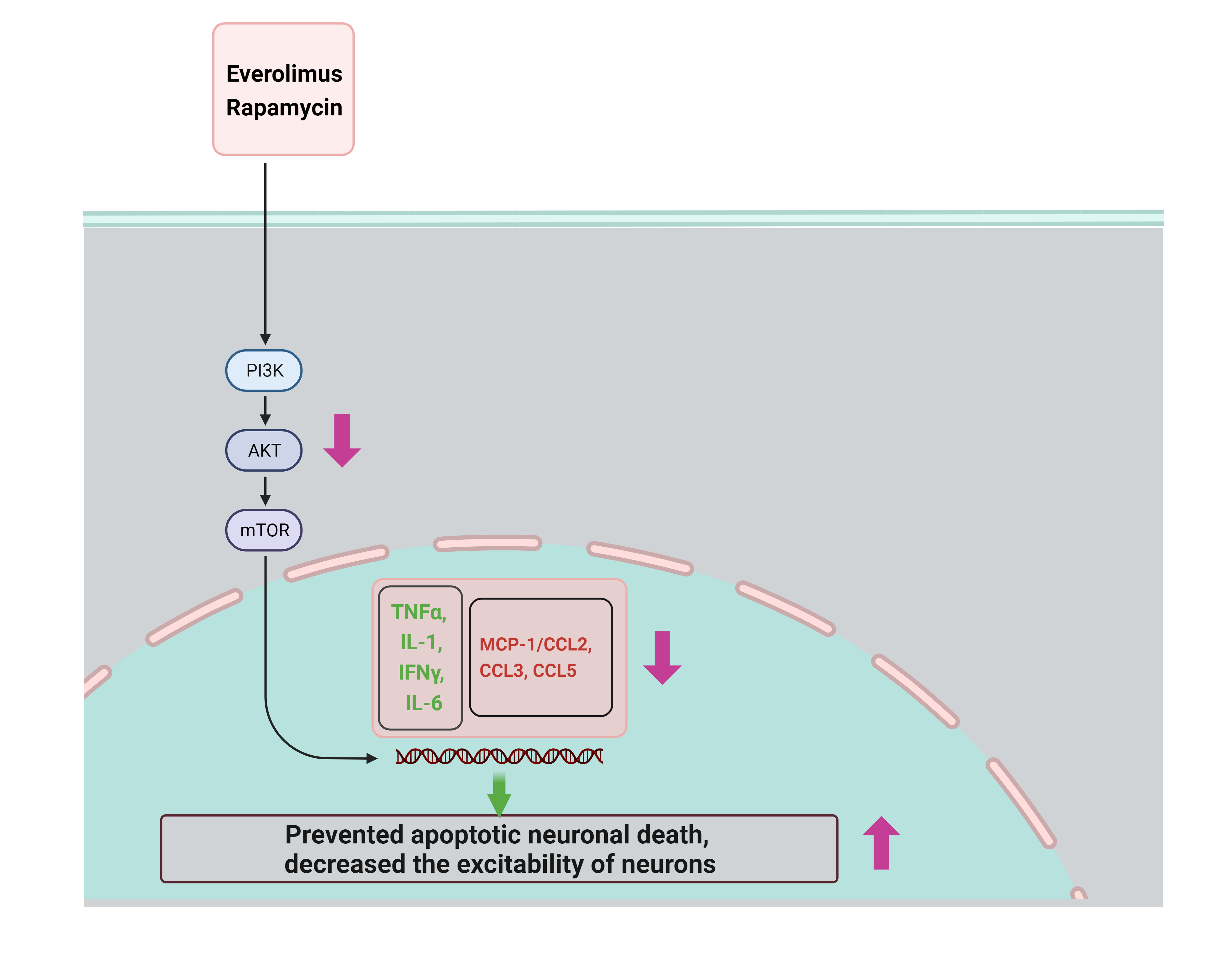


**Fig. S2** Antipsychotic drugs significantly ameliorated schizophrenic-like behavior in ketamine-induced rats via activating the PI3K/AKT/mTOR signaling pathways and increasing the mRNA expression of NRG1 and ErbB4 in the hippocampus of rats with schizophrenia.


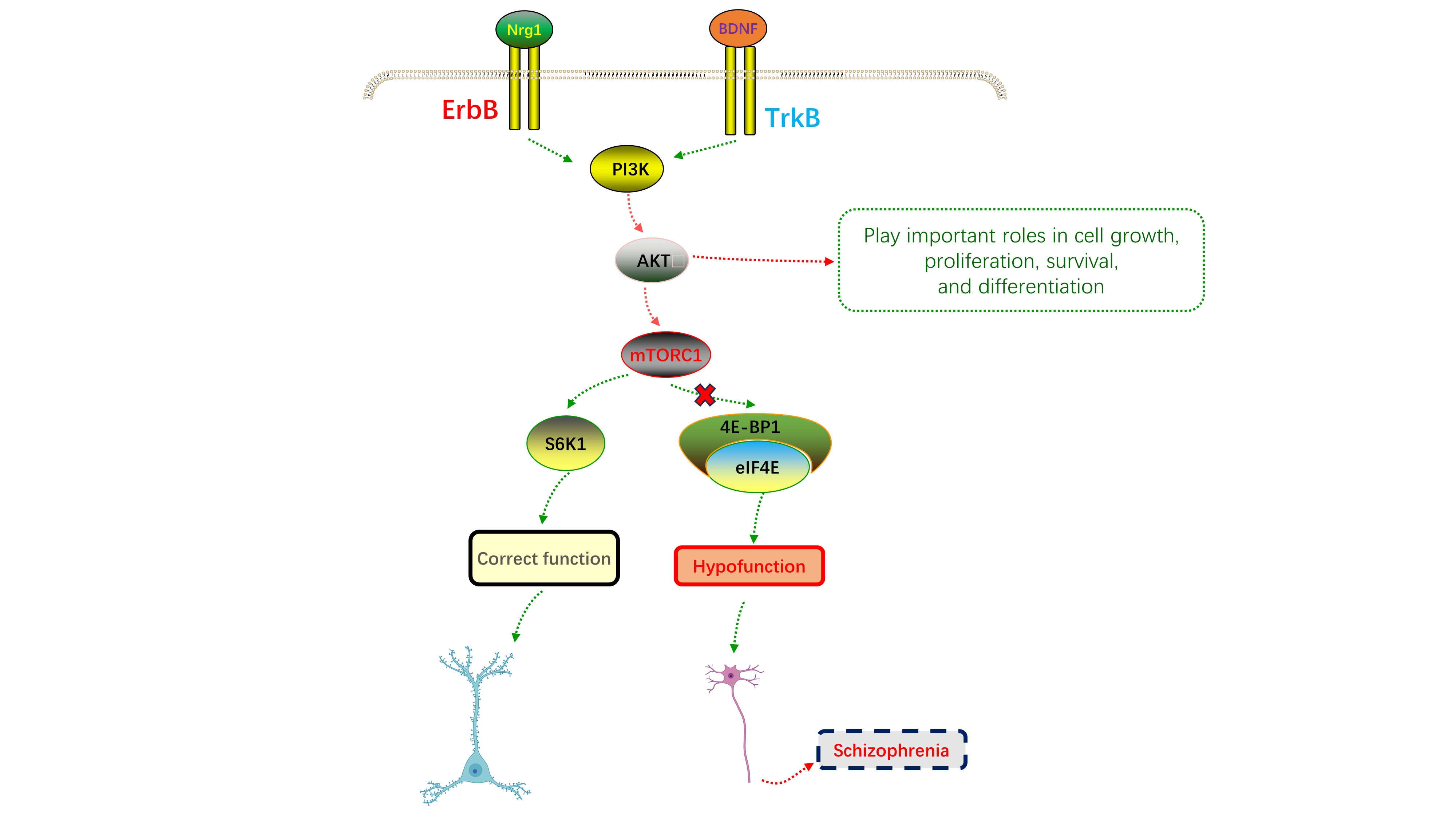

Supplement: pyae010_suppl_Supplementary_Material [file pyae010_suppl_supplementary_material.docx]
