# Supplementary figures and images for: PI3K-AKT/mTOR Signaling in Psychiatric Disorders: A Valuable Target to Stimulate or Suppress?
Source: Int J Neuropsychopharmacol. 2024 Feb 14;27(2):pyae010. doi: 10.1093/ijnp/pyae010 (PMC10888523; doi:10.1093/ijnp/pyae010)

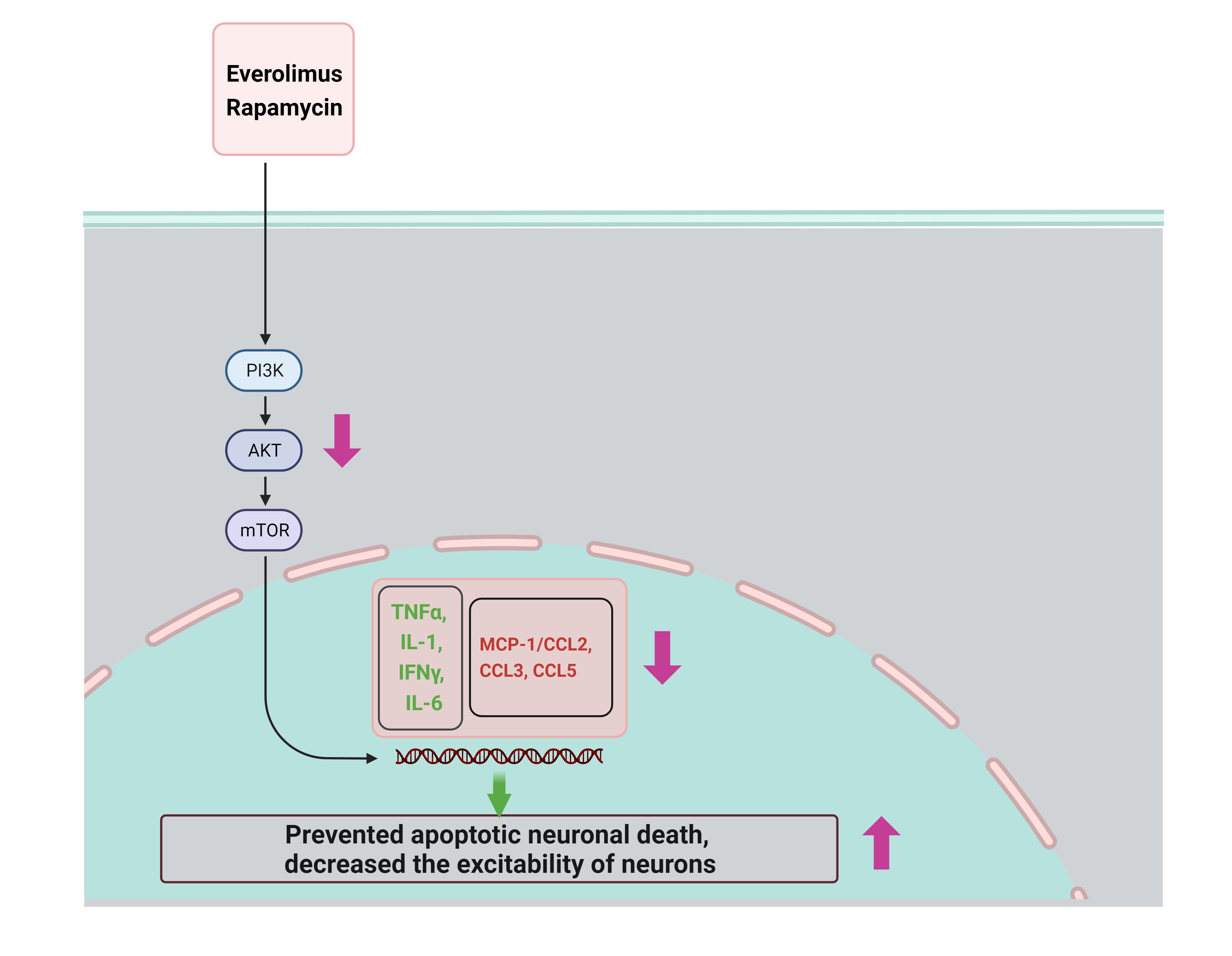

Supplement: pyae010_suppl_Supplementary_Figure_S1 [file pyae010_suppl_supplementary_figure_s1.jpeg]

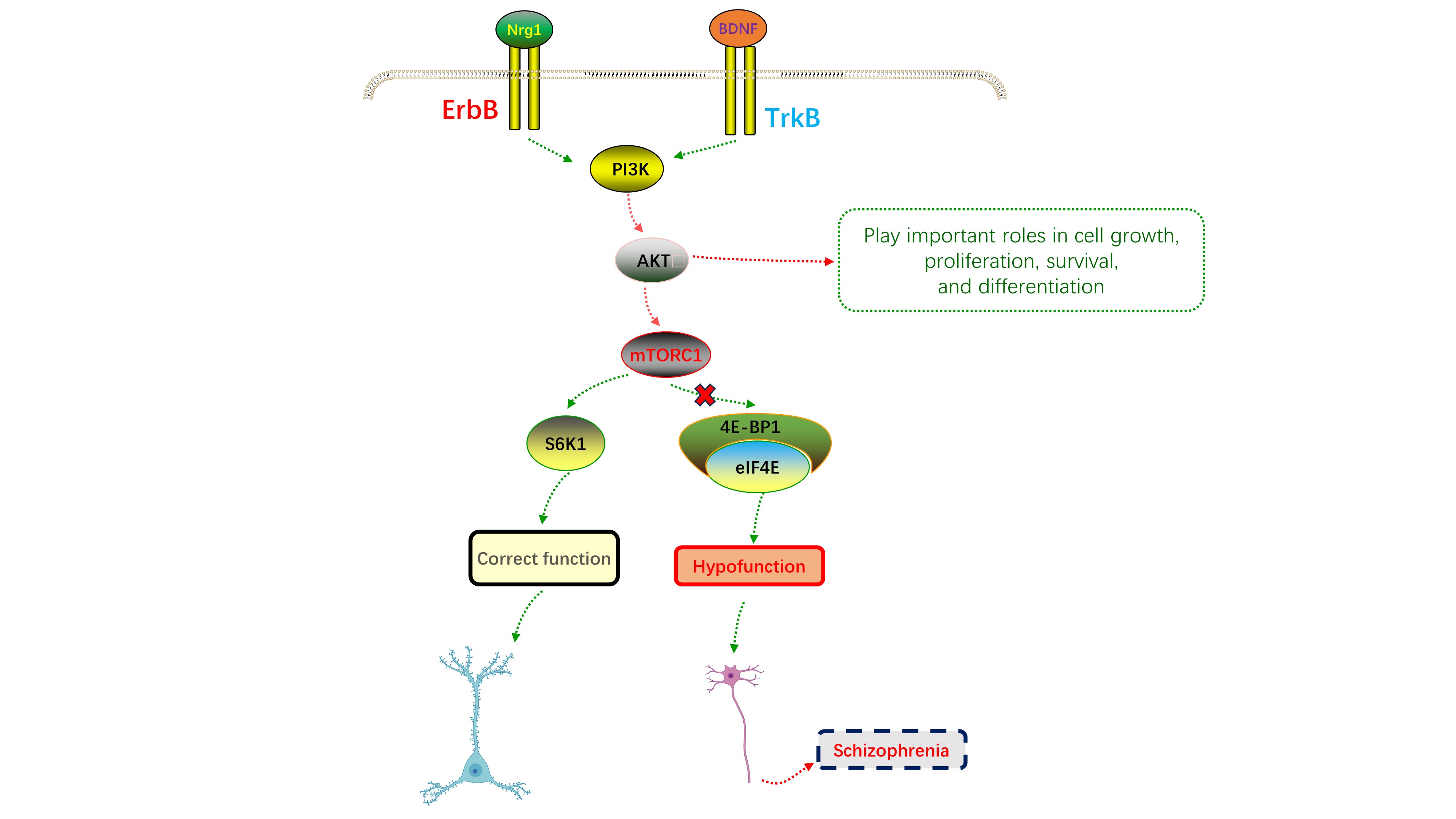

Supplement: pyae010_suppl_Supplementary_Figure_S2 [file pyae010_suppl_supplementary_figure_s2.jpeg]
